# Anti-SARS-CoV-2 Antibody Level among Renal Transplant Recipients: A Case Report from Nepal

**DOI:** 10.1155/2022/2889501

**Published:** 2022-01-10

**Authors:** Kamal Ranabhat, Bhuvan Saud, Saroj Adhikari, Suraj Bhattarai, Rojan Adhikari, Bhoj Raj Luitel, Mahesh Raj Sigdel

**Affiliations:** ^1^Institue of Medicine, Tribhuvan University, Kathmandu, Nepal; ^2^Ministry of Health and Population, Kathmandu, Nepal; ^3^Department of Medical Laboratory Technology, Janamaitri Foundation Institute of Health Sciences, Lalitpur, Nepal; ^4^National Trauma Center, Ministry of Health and Population Kathmandu, Nepal; ^5^Global Institute for Interdisciplinary Studies, Kathmandu, Nepal; ^6^Shahid Dharma Bhakta National Transplant Center, Bhaktapur, Nepal

## Abstract

Globally, SARS-CoV-2 has caused significant public health burden, mainly in patients with underlying comorbidities including both communicable and noncommunicable diseases. Solid organ transplant recipients under immunesupressive medication are also amongst the high risk group. There is only sparse data on immunity against SARS-CoV-2 infection among renal transplant recipients. In this case report, we present the level of anti-SARS-CoV-2 antibody of three kidney transplant recipients after vaccination against COVID-19 virus. All three cases had received two doses of Oxford-AstraZeneca COVID-19 vaccine AZD1222 (ChAdOx1). Serological analysis showed protective level of circulating antibodies in the blood of all three cases. Although two out of three patients in the study acquired COVID-19 infection after immunization, they recovered with mild clinical course. Hence, we conclude that despite immune-suppressed status of transplant recipients, COVID-19 vaccination could protect them against severe illness.

## 1. Introduction

Coronavirus disease 2019 (COVID-19) is caused by Severe Acute Respiratory Syndrome Coronavirus-2 (SARS-CoV-2). The virus causes significant morbidity and mortality in individuals with underlying clinical conditions. As of 8th January 2022, more than 302 million positive cases and 5.4 million deaths have been reported globally [1].Generally, pathogenic microorganisms cause pathological manifestations in host system which depends on the host immune response and virulence factors of the infectious agents. Human body activates complex immune mechanism by which it provides protection from the invading foreign substance [2]. In SARS-CoV-2 infection, patients produce virus-specific B and T cells and then initially produce Immunoglobulin M (IgM) and later Immunoglobulin G (IgG) antibodies which are readily detected in serum [3, 4]. Generally, patients with chronic diseases are highly susceptible to the infection. Higher mortality rate in such group may be due to dysregulation of immune system. Studies have suggested that haemodialysis and kidney transplant patients are at high risk of morality from SARS-CoV-2 with 20% mortality rate [5, 6]. Kidney transplant recipients are advised to take immune-suppressive drugs (calcineurin inhibitors, antiproliferative agents, rapamycin inhibitors, and corticosteroids) in order to prevent rejection by the host immune system. Lowered immunity makes such person susceptible to infectious agents [7].

Globally, as of 7th January 2022, 137 different COVID-19 vaccines are in various phases of clinical trials, while 194 are in the preclinical phase; emergency-use approved vaccines are recommended prioritizing people with chronic diseases, with certain clinical conditions and old age [8]. Studies have reported that vaccines show weak immunogenicity among kidney transplant recipients as they do not produce antibodies effectively [9, 10]. Moreover, live or replication component vaccines are contraindicated in transplant recipients [11]. In contrast, some studies have noted that solid organ transplant recipients achieve adaptive immune response similar to immune-competent individuals [12]. A study conducted among kidney transplant recipients in France has revealed that 10.8% of transplant recipients developed antibodies after 28 days of receiving COVID-19 vaccine and that multiple booster doses might be required to reach effective level [13].

In Nepal, immune response to SARS-CoV-2 in immune-compromised individuals, mainly in renal transplant recipients has not been reported till date and very less is known about neutralizing antibody response in them after vaccination. In this study, we estimated circulating anti-SARS-CoV-2 antibody level after vaccination in three renal transplant patients who are concurrently receiving immune-suppressive drugs. It was aimed to understand the humoral immune response in the recipients and the need for additional booster doses of COVID-19 vaccine in such patients.

## 2. Case Presentation

### 2.1. Patient 1

A 40-year-old overweight male had kidney transplant in 2008 (13 years ago). He received first dose of ChAdOx1 nCoV-19 vaccine (AZD1222) against SARS-CoV-2 on 16 April 2021 and the second dose on 21 June 2021. A quantitative serological test was performed on 26 June 2021 to detect anti-SARS-CoV-2 antibody level in his blood by Chemiluminescent immunoassay (CLIA) test as per the manufacturer's instructions The test result was 18.15 cutoff index (test positive if value is >1.) Later, on 19 October 2021, the man was admitted to the hospital with mild illness, having fever (101.5°F), body ache and loss of smell. Real-time polymerase chain reaction (RT-PCR) test for COVID-19 was found positive and became negative only after 23 days of first diagnosis. Other clinical laboratory parameter results are shown in Table 1.

### 2.2. Patient 2

A 38-year-old male had renal transplantation in 2005 (16 years ago). He received the first dose of ChAdOx1 nCoV-19 vaccine (AZD1222) in April 2021 and the second dose in June 2021 (7 weeks later). His anti-SARS-CoV-2 serum antibody level was tested on 25 June 2021 which was 54.5 by enzyme-linked immunosorbent assay (ELISA) where test is positive if value is >11.0. On 24 November 2021, he experienced mild symptoms of COVID-19 such as fever, malaise, sore throat, and loss of smell and taste. He was admitted to the hospital and was found positive for COVID-19 by RT-PCR test. He tested negative for COVID-19 virus gene detection after one month. Before natural infection, the patient's other clinical laboratory reports are listed in Table 1.

### 2.3. Patient 3

A 41-year-old male had undergone kidney transplantation in 2006 (15 years ago). He took the first dose of ChAdOx1 nCoV-19 vaccine (AZD1222) on 1 June 2021 and the second dose on 8 July 2021. He did not experience COVID-19 infection. His blood was tested for anti-SARS-CoV-2 antibody level on 28 July 2021. Chemiluminescent immunoassay (CLIA) test report showed that anti-SARS-CoV-2 antibody level was 5.91 IU/ml, where test is positive if the value ranged >0.80 IU/ml. Other laboratory parameters are shown in Table 1.

## 3. Discussion

According to the report published by Shahid Dharma Bhakta National Transplant Center, Bhaktapur, annually, 3000 cases of kidney failure are reported in Nepal and out of them more than 350 kidney transplants are performed [14]. Kidney transplant recipients are at higher risk of microbial infection due to immunosuppressive status. In this case series, we present clinical and laboratory findings of three kidney transplant recipients in Nepal who were vaccinated against SARS-CoV-2 infection. The total leucocyte count and differential leucocyte count of all three cases were normal indicating that the therapeutic indices of the immune-suppressive medications had been maintained. Two recipients got infected by SARS-CoV-2 after completing two doses of vaccine but only presented mild symptomatic illness and successfully recovered without requiring critical care support. Mean age of transplant recipients was 39.6 (range, 38–41 years) and all three were male. Neutralizing antibody was developed in all three cases with significant antibody level. However, since the duration of protection from the vaccine is not clearly known yet, there is need for frequent follow-up for neutralizing antibody levels in such patients which is subjected to drop due to immune-suppressive medication. The result of this study is also different from other studies which reveal that very few individuals who received organ transplant developed anti-SARS-CoV-2 antibody [15, 16]. Kidney transplant recipients are at higher risk of developing COVID-19 severity if the recipients have comorbid diseases (obesity, diabetes, asthma, and chronic pulmonary disease), particularly diabetes and hypertension which are recognized as one of the main factors responsible for severity [16].

In this study, seroconversion occurred in all three transplant patients in an effective level. Studies have suggested that only 4-48% of renal transplant recipients have detectable anti-spike IgGs following two doses of COVID-19 vaccine [17]. In a study conducted among immune-suppressed solid organ transplant recipients from USA, who received Ad26.COV2.S (Janssen COVID-19 vaccine), out of 12 participants, only two (16%) had mounted detectable amount of antibody level [18]. A study from France conducted among kidney transplant recipients after administration of two doses of BNT162b2 mRNA COVID-19 vaccine (Pfizer-BioNTech) found that 5.7% of the patients developed anti-spike antibodies [19]). Furthermore, a case control study conducted in Germany to evaluate the immune response after vaccination in kidney transplant recipients with BNT162b2 mRNA COVID-19 vaccine (Pfizer-BioNTech) found that only 22% of the recipients were positive for anti-SARS-CoV-2 IgG antibodies. The titers were also significantly lower than the control group of heath-care workers. Anti-SARS-CoV-2 IgG antibodies were detected in 100% of the participants in the control group following vaccination [6]. The clinical outcome of the vaccinated renal transplant recipients in our study was favorable. Although two of the three patients got infected with COVID-19, both recovered and are stable. In a study conducted in India among four renal transplant recipients, all of them got infected with COVID-19 despite being vaccinated full doses of ChAdOx1 nCoV-19 vaccine (AZD1222); one of them died, two were admitted on ventilator at the time of the study, and another patient recovered and was stable at home [20].

Tacrolimus induces neutropenia [21] while mycophenolate mofetil leads to reduction of B-lymphocyte count [22]. It is important to maintain the circulating level of these drugs within therapeutic ranges to maintain health and prevent graft rejection. Both renal function tests and complete blood count profile are within normal range and the patients do not present any comorbidity which reflects that the immune-suppressant regimens are maintained within their therapeutic indices. The production of protecting level of humoral immune response against COVID-19 virus after vaccination in the study cases may be attributed to the maintained drug levels.

The limitations of this study are that antibody levels were not estimated periodically nor the antibody level was tested after first dose of vaccine administration. Another limitation is that only ChAdOx1n CoV-19 (AZD1222) vaccinated individuals were enrolled in this study but the immune response to other types of COVID-19 vaccines might be different. Also, periodic reports of serum level of the immune-suppressive drugs could not be obtained which would have better justified the immune status of the participants in the study. For better evidence, similar serological studies should be conducted in large scale to understand the immunogenicity of newer vaccines in the immune-compromised population, the results of which would help the government and concerned authorities to take prompt action.

## 4. Conclusion

Protective level of humoral immune response against SARS-CoV-2 has been shown after administration of two doses of ChAdOx1 nCoV-19 (AZD1222) vaccine in renal transplant recipients. Routine monitoring of anti-SARS-CoV-2 circulating antibody as well as immune-suppressive drug level is recommended to understand the immune status of transplant recipients and the immunogenicity of newer vaccines in them.

## Figures and Tables

**Table 1:** Different variables of the three renal transplant recipients.

Clinical variables	Patient 1	Patient 2	Patient 3
Age	40	38	41
Gender	Male	Male	Male
Transplant performed	2008	2005	2006
Current medications	Mycophenolate sodium 360 mg and tacrolimus 0.5 mg	Mycophenolate mofetil 500 mg and cyclosporine 25 mg	Mycophenolate mofetil 500 mg and tacrolimus (1 mg∗ and 0.5 mg∗∗)
Body mass index	Overweight	Normal	Overweight
Comorbidities	No	No	Hypertension
1st dose COVID vaccine	16 April 2021	21 April 2021	01 June 2021
2nd dose COVID vaccine	21 June 2021	08 June 2021	08 July 2021
Serum creatinine (mg/dl)	1.3	1.4	1.2
Serum urea (mg/dl)	41.0	16.96	21.0
Sodium (Na^+^) mmol/l	136	139.0	143.0
Potassium (K^+^) mmol/l	4.8	3.3	4.0
Hemoglobin (g/dl)	15.8	12.6	12.7
RBC (million/mm^3^)	5.48	4.64	4.76
WBC (cells/mm^3^)	7900	8400	12500
Differential cell count			
Neutrophil (%)	78	74	77
Lymphocyte (%)	16	21	21
Monocyte (%)	04	4	02
Eosinophil (%)	02	1	0
Basophil (%)	0	0	0
RT-PCR for COVID-19	Positive	Positive	Not tested
COVID-19 sign and symptoms	Fever, body ache, and loss of smell	Fever, malaise, sore throat, and loss of smell and taste	Unknown
Anti-SARS-CoV-2 ab (method)	18.15 COI (ECLIA)	54.5 (ELISA)	5.91 U/ml (ECLIA)
Outcome	Recovered from COVID-19	Recovered from COVID-19	Stable

*Note*. ∗Morning dose; ∗∗evening dose. NR: normal range; RT-PCR: reverse transcription polymerase chain reaction; ELISA: enzyme-linked immunosorbent assay; ECLIA: electrochemiluminescence immunoassay; ab: antibody; COI: cutoff index.

## Data Availability

The data used to support the findings of this study are available from the corresponding author upon request. The authors agree that anyone interested can have access to the raw data from the research upon request to the authors. The data will be provided to anyone based on two conditions: upon use of the data, the authors should be acknowledged and also the paper needs to be cited. An agreement in the aforementioned situation will be reached.
